# The impact of measurement error in modeled ambient particles exposures on health effect estimates in multilevel analysis: A simulation study

**DOI:** 10.1097/EE9.0000000000000094

**Published:** 2020-05-27

**Authors:** Evangelia Samoli, Barbara K. Butland, Sophia Rodopoulou, Richard W. Atkinson, Benjamin Barratt, Sean D. Beevers, Andrew Beddows, Konstantina Dimakopoulou, Joel D. Schwartz, Mahdieh Danesh Yazdi, Klea Katsouyanni

**Affiliations:** a Department of Hygiene, Epidemiology and Medical Statistics, Medical School, National and Kapodistrian University of Athens, Athens, Greece; b Population Health Research Institute, St George’s, University of London, London, United Kingdom; c MRC Centre for Environment and Health, King’s College London, London, United Kingdom; d National Institute for Health Research Health Protection Research Unit (NIHR HPRU) in Health Impact of Environmental Hazards, King’s College London, London, United Kingdom; e Department of Environmental Health, Harvard School of Public Health, Boston, Massachusetts; f Department of Epidemiology, Harvard School of Public Health, Boston, Massachusetts; g School of Population Health and Environmental Sciences and MRC Centre for Environment and Health, King’s College London, London, United Kingdom

**Keywords:** Health effects, Measurement error, Modeled air pollution, Particulate matter

## Abstract

Various spatiotemporal models have been proposed for predicting ambient particulate exposure for inclusion in epidemiological analyses. We investigated the effect of measurement error in the prediction of particulate matter with diameter <10 µm (PM_10_) and <2.5 µm (PM_2.5_) concentrations on the estimation of health effects.

**Methods:**

We sampled 1,000 small administrative areas in London, United Kingdom, and simulated the “true” underlying daily exposure surfaces for PM_10_ and PM_2.5_ for 2009–2013 incorporating temporal variation and spatial covariance informed by the extensive London monitoring network. We added measurement error assessed by comparing measurements at fixed sites and predictions from spatiotemporal land-use regression (LUR) models; dispersion models; models using satellite data and applying machine learning algorithms; and combinations of these methods through generalized additive models. Two health outcomes were simulated to assess whether the bias varies with the effect size. We applied multilevel Poisson regression to simultaneously model the effect of long- and short-term pollutant exposure. For each scenario, we ran 1,000 simulations to assess measurement error impact on health effect estimation.

**Results:**

For long-term exposure to particles, we observed bias toward the null, except for traffic PM_2.5_ for which only LUR underestimated the effect. For short-term exposure, results were variable between exposure models and bias ranged from −11% (underestimate) to 20% (overestimate) for PM_10_ and of −20% to 17% for PM_2.5_. Integration of models performed best in almost all cases.

**Conclusions:**

No single exposure model performed optimally across scenarios. In most cases, measurement error resulted in attenuation of the effect estimate.

## Abstract 2

Supplemental Digital Content is available in the text.

**What this study adds**

Epidemiological studies of the health effects of long- and short-term exposure to outdoor particulate air pollution that utilize modeling techniques to derive pollution exposures will generally underestimate the magnitude of the associations (with overestimates in some cases). These biases are not trivial and should therefore be considered when assessing the evidence from epidemiological studies in policy evaluation and health impact assessment exercises. This study also suggests no single air pollution modeling method is optimal and further work on the integration of models to maximize performance is advisable.

## Introduction

The difficulty in defining ambient particles, given that their chemical and physical properties vary by time period, location, sources and meteorology, makes the understanding of measurement error implications on health effects estimation even more important than gaseous pollutants unless we assume equitoxicity. As mentioned in our joint article,^1^ measurement error in air pollution exposure estimates and the resulting impact on the estimation of health effects has attracted attention in recent years.^2^ Szpiro et al^3^ showed that better exposure prediction by land-use regression (LUR) models does not necessarily result in less bias in the health effect estimate following long-term exposure. A review^4^ concluded that measurement error mostly negatively biased the effect estimates and increased standard errors, especially when exposure concentration was modeled with low spatial and temporal resolution for a spatially variable pollutant.

Within the framework of the “Comparative evaluation of Spatio-Temporal Exposure Assessment Methods for estimating the health effects of air pollution” (STEAM) project, we assessed the impact of measurement error in spatiotemporal exposure predictions developed for greater London for 2009–2013 on the health effect estimate in a mixed Poisson model that allows for the simultaneous estimation of effects following short- and long-term exposure.^5^ We previously^6^ evaluated the impact of several scenarios and indicated that measurement error in NO_2_ and PM_10_ resulted mostly in the attenuation of effect estimates for both short- and long-term exposure. In this article, we present the results of an extensive simulation study to address the impact of measurement error from spatiotemporal predictions of PM_10_ and PM_2.5_ concentrations from various exposure assessment models on the effect estimates of daily mortality and hospital admissions due to cardiovascular diseases (CVD).

## Methods

We set up simulations for a sample of 1,000 lower super output areas (LSOAs, a small geographic area) in the Greater London area^7^ informed by correlation coefficients and variance ratios estimated from validation datasets for 2009–2013, which compare modeled pollutant data with measurements from the extensive London network of fixed-site monitors. We simulated from reported coefficients for two different outcomes: all-cause mortality and CVD hospital admissions, driven by the need to assess differential behavior depending on the prevalence and the variability of the outcome and the range of the effect estimate. Simulations were based on concentration-response functions (CRF) that varied in magnitude to allow for the assessment of a range of situations that have been reported in the literature. For each scenario, 1,000 simulations were run.

### Measurements from fixed site monitors and enhanced PM_2.5_ database

We constructed a database of ambient particles (24-hour average PM_10_ and PM_2.5_) concentrations including all measurements from sites within the M25 orbital highway, during the years 2009–2013, obtained from the London Air Quality Network,^8^ Air Quality England,^9^ and the Automatic Urban and Rural Network.^10^ For PM_10_, we compiled data from 115 sites while PM_2.5_ data were available only from 33 sites. To inform LUR and machine learning models and the validation datasets used in the simulations, we needed a larger PM_2.5_ database, hence we enhanced the available data based on a modeling approach.

Briefly, at each PM_10_ monitoring location, we fit models for combining PM_2.5_ predictions from a generalized additive model (GAM) and a random forest approach,^11^ both of which incorporated seasonality trend, concurrent measurements of other pollutants, and meteorological variables. The predictions from each model were entered as spline functions in a new GAM. The 10-fold cross-validation adjusted *R*^2^ of the combined model was 98.9%.

The final data included information from 37 urban/suburban sites for PM_10_ and 32 for PM_2.5_, and from 65 roadside/kerbside for PM_10_ and 60 for PM_2.5_.

### PM exposure models

We developed spatiotemporal LUR and dispersion models to estimate the particles’ concentration at the postcode centroid level which were then averaged to produce concentrations at LSOA level. LUR models provide estimates at specific geographical point coordinates (e.g., the postcode centroid) while dispersion estimates provide exposure maps at a 20 × 20 m grid and we subsequently applied bilinear interpolation to estimate concentrations at a certain location.^1^ For PM_2.5_, we further incorporated satellite measurements and applied three machine learning algorithms that were combined in a GAM to produce spatiotemporal concentrations at a 1 × 1 km grid.

### Land-use regression models

We developed spatiotemporal semiparametric models where the measurement of the particles at location *i* on day *t* is modeled as a combination of smooth functions reflecting the nonlinear effects of several temporal covariates (daily mean temperature, daily mean wind direction, daily mean barometric pressure, variable for day count, accounting for trends within each year) and a spatial covariate (inverse distance of monitoring sites to the nearest major road).We included indicator variables for different years (reference category was 2009), daily mean relative humidity, daily mean wind speed, total traffic load in a buffer of 100 m around each monitoring site and total length of major roads in a buffer of 300 m around each monitoring site. A bivariate smooth function of geographical coordinates accounting for residual correlation between locations was included.

### Dispersion models

The Community Multiscale Air Quality urban (CMAQ-urban) model^12,13^ combines emissions data with the Weather Research and Forecasting meteorological model^14^ and the Community Multiscale Air Quality (CMAQ) model (v5.0.2),^15^ which has been coupled to the Atmospheric Dispersion Modelling System roads model (v4).^16^ Driven by meteorological fields from the WRF model, the CMAQ-urban model outputs hourly air pollution concentrations at high spatial resolution and predicts air pollution concentrations at points spaced 20 m apart across the STEAM area. To provide a concentration at the fixed sites, we used bilinear interpolation of the nearest 20 m points.

### Machine learning algorithms for satellite-based models

Only for the exposure assessment of PM_2.5_, we applied three machine learning algorithms that incorporated all the available spatiotemporal covariates along with satellite measurements on aerosol optical depth (AOD) using the MAIAC algorithm for MODIS. Specifically we used measurements from both the Aqua and Terra Satellite, with data on population density, cloudiness, barometric pressure, wind direction, wind speed, dewpoint temperature, temperature, land use type, distance to water, distance to Heathrow, inverse of the height of the planetary boundary layer, normalized difference vegetation index, traffic counts, and day of the year (with a sine and cosine function). Machine learning algorithms are prediction algorithms that train on a subset of the data, predict on held out data, and choose training parameters that maximize predictive power in the held out, testing data. By design, they can incorporate highly nonlinear and highly interactive models, without prespecifying which variables are nonlinear, what the nonlinearity looks like, and which variables interact.

We trained three models (random forest,^11^ neural network,^17^ gradient boosting^18^) to predict PM_2.5_ separately from Aqua AOD and Terra AOD, therefore six models in total. Training was based on a grid search of hyperparameters for each learner, using internal cross-validation (CV) and mean square error as the criteria for selection. The neural network included a Least Absolute Shrinkage and Selection Operator on the variables to reduce overfitting. We then combined the six individual predictions of the output of the methods in a GAM using unconstrained smooth functions and a smooth function for longitude and latitude.

### Hybrid models

By weighing the individual methods’ possibly different performance along the concentration range of the pollutants, the combination of different methods may result in less measurement error and subsequently less bias in the health effect estimates. We therefore applied the following hybrid models depending on availability of approaches:

Hybrid 1: For PM_10_ and PM_2.5_, we constructed a combined LUR-dispersion model by incorporating into the LUR a smooth function of the daily predictions from the dispersion model.

Hybrid 2: For PM_10_ and PM_2.5_, a GAM approach was applied to combine predicted pollutant concentrations from the developed spatiotemporal LUR and CMAQ-urban dispersion models. The GAM was developed by fitting two corresponding splines of the predicted variables (LUR and CMAQ). For the LUR, we used 10-fold cross-validated predictions.

Hybrid 3: In the case of PM_2.5_, the hybrid model 2 was extended to include a smooth function of the predictions from the combined machine learning methods.

### Simulations set-up

The set-up of the simulations has been presented in the companion paper.^1^ Briefly, we (1) sample 1,000 LSOAs with their coordinates from the study area; (2) For this sample of 1,000 LSOAs, we simulated “true” daily pollutant concentrations (X*) informed by either the urban/suburban or the kerbside/roadside fixed sites assuming that differential measurement error occurs by site type. Temporal correlation and the spatial variation, as estimated by a covariance model fitted to the empirical semivariogram, were incorporated in the “true” surface that was also adjusted for instrument error in the monitor measurements; (3) We simulated a daily health outcome (Y) over 2009−2013 from the “true” pollutant data using CRF from the literature (eAppendix, Table S1; http://links.lww.com/EE/A88) based on a simple multilevel Poisson regression model, with a random intercept per LSOA, where the effect of short-term exposure is estimated by the coefficient corresponding to the daily time-series and the effect of long-term exposure to the coefficient of the average over the period exposure; (4) We added to the “true” daily pollutant concentrations measurement error informed by the validation data at the fixed sites that provided estimates of the spatial and temporal correlations and variance ratios. A new pollution variable (Z) corresponding to each exposure method was created; (5) We analyzed the association between each health outcome (Y) and new pollutant (Z) and estimated the two coefficients denoting the effect following short- and long-term exposure and their standard errors; (6) We ran 1,000 simulations and assessed the results in terms of bias (mean difference between true and estimated effect estimate), statistical power (percentage of simulations where the effect estimate is statistically significant at the 5% level) and coverage probability (% of simulations where the 95% confidence interval [CI] contains the true CRF).

All analyses were run in R version 3.4.3 (http://www.R-project.org/, 2017) using the libraries *mgcv*, *randomForest*, *Hmisc*, *lme4*, *MASS*, and *foreign*. In GAM, the default generalized cross-validation criterion (GCV) was used for the choice of the smoothing parameter as defined in the *mgcv* library.

## Results

Table 1 presents the spatial and temporal correlation coefficients between the “true” and modeled concentrations and their corresponding variance ratios (modeled over “true”) as provided by validation data. These inform the simulations of particulate concentrations for each assessment method from the “true” exposure surface and define the scenarios presented in Tables 2 and 3. Temporal correlations were larger compared with spatial ones. Of the 16 variance ratios (eight spatial and eight temporal) calculated for PM_10_ and for PM_2.5_, five per pollutant deviated from 1 by less than 10%, and these were mostly temporal. The LUR consistently displayed the lowest and the dispersion model the highest temporal variance ratio.

**Table 1.**
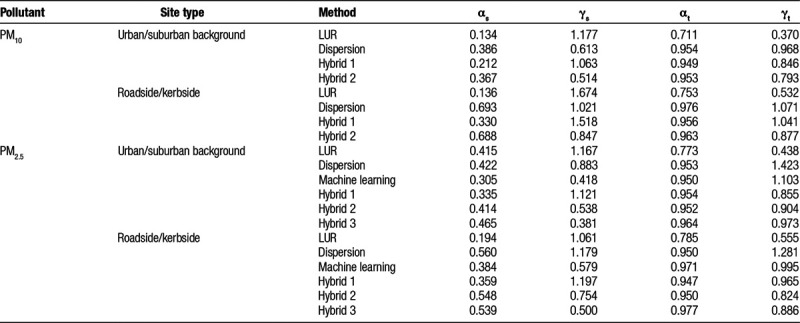
Estimates of spatial and temporal correlation coefficients (α_s_ and α_t_) and variance ratios (γ_s_ and γ_t_).

**Table 2.**
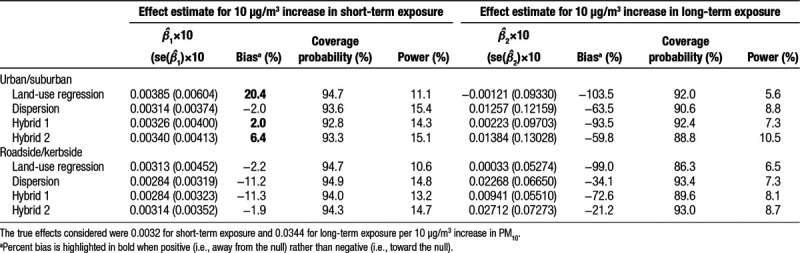
Simulations’ results for the association between all-cause mortality and PM_10_.

**Table 3.**
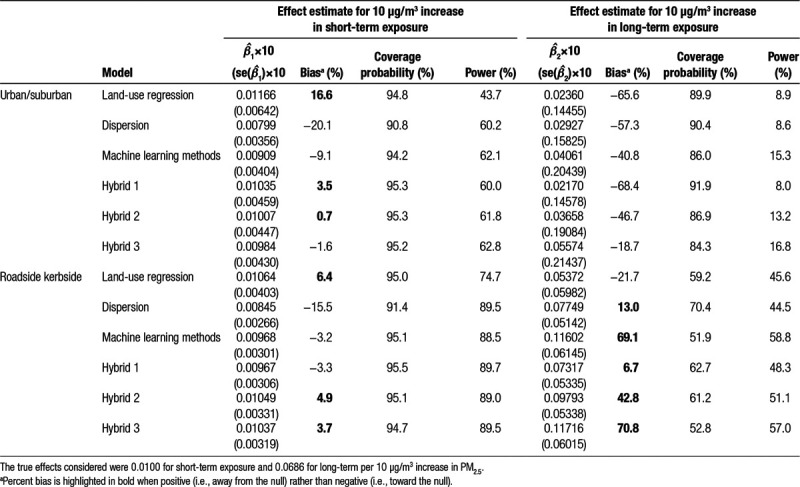
Simulations’ results for the association between all-cause mortality and PM_2.5_.

Table 2 presents the simulations results for the associations between PM_10_ and total mortality. CVD hospital admissions results are presented in eAppendix, Table S2; http://links.lww.com/EE/A88. Regarding long-term exposure results, all models irrespective of outcome, method, and monitor type displayed bias toward the null ranging from −21% to −104%. For both total mortality and CVD admissions, the best-performing model was hybrid 2 with biases of −60% and −48% for urban/suburban monitors and −21% and −26% for roadside/kerbside monitors. Coverage probabilities were high for mortality but very low for CVD admissions that were simulated based on a much larger CRF as compared to mortality. Statistical power was generally low.

Results for mortality effects following short-term exposure displayed negative (i.e., towards the null) bias for roadside/kerbside monitors ranging from −2% (hybrid 2) to −11% (hybrid 1) and variable bias for urban/suburban monitors: relatively small for the dispersion (−2%), the hybrid 1 (+2%), and the hybrid 2 (+6.4%) and larger for the LUR (+20%). Coverage probabilities ranged from 93% to 95% with power between 11% and 15%. Hospital admission analysis provided similar results but with higher statistical power. For both outcomes, the best-performing models were the hybrid 2 model for kerbside/roadside concentrations and the dispersion prediction when considering urban/suburban sites.

Table 3 presents the simulation results for the associations between PM_2.5_ and total mortality, while eAppendix, Table S3; http://links.lww.com/EE/A88, presents results for CVD hospital admissions. Results were more variable in the direction of bias compared with PM_10_ results. For the long-term results, considerable negative bias (i.e., toward the null) was exhibited for all models under the urban/suburban characterization of the simulated “true” exposure, with the hybrid 3 model having the smallest bias (−19% for mortality and −21% for CVD admissions). For the kerbside/roadside sites, positive bias (i.e., away from the null) ranging from +7% to +73% was displayed for all except the LUR (−22% for mortality; −6% for CVD) predictions. The best-performing model in terms of the magnitude of the bias being hybrid 1 (incorporating dispersion estimates into LUR) for total mortality and the LUR model for the CVD admissions. For short-term results, biases, though variable in direction, were generally small ranging from −20% to +17% across outcomes and site-type. Coverage probabilities for PM_2.5_ were generally high, except for long-term exposure based on roadside/kerbside sites. Statistical power was highest for short-term exposure within roadside/kerbside scenarios (>74%) and lowest for long-term exposure within urban/suburban scenarios (<25%). Validation statistics (eAppendix, Table S4; http://links.lww.com/EE/A88) support better performance of the hybrid models.

## Discussion

Our simulations indicated bias toward the null for most scenarios, except in the case of kerbside/roadside PM_2.5_ that showed a bias away from the null for long-term exposure. For PM_10_ under most scenarios, the hybrid 2 model combining predictions from LUR and dispersion methods exhibited the smallest bias. The combination of methods under Hybrid model 3 performed best for urban/suburban PM_2.5_ for both outcomes, while for kerbside/roadside, the machine learning algorithms provided the most accurate estimate for short-term exposure but not for long-term exposure, where the best model appeared to be hybrid 1 for mortality and LUR for CVD admissions. Our approach simulates situations in which the spatial and temporal correlation coefficients and variance ratios relating the “pseudo” modeled and “true” data mirror those estimated from the validation datasets (including adjustment for instrument error in the measurements) and it is the correlation coefficients and variance ratio that we are testing out in our simulations.

In line with Butland et al,^6^ we find that as correlation gets smaller and the variance ratio larger the bias toward the null is increased, while bias away from the null was noted for high correlations and small variance ratios. The error in the “modeled” exposures is a combination of classical and Berkson that is not distinguishable, although larger Berkson-like error is expected in methods with smaller variance ratio. This scenario in most cases corresponds to Hybrid models but is not consistent across the temporal and spatial terms.

Although results from PM_2.5_ are more variable, combination of methods performed better than individual ones. Bias toward the null for long-term effects was observed for all methods for urban/suburban monitors, while bias away from the null was observed for kerbside/roadside PM_2.5_ for five out of six methods. However for short-term exposures, biases though varying in direction were relatively small.

The optimal performance of combinations of methods under nearly every scenario may be attributed to potentially better capture of different characteristics of the particles’ distribution and composition. For example, the combination of several machine learning algorithms may perform better in traffic-related PM_2.5_ as it may be more flexible in capturing a variety of interactions between covariates and their shapes and hence better capture variability of levels near traffic. In all cases, the Hybrid models attributed more degrees of freedom to estimates derived from the dispersion models, then to machine-learning predictions and less to those from LUR.

Previous air pollution exposure research^19,20^ mainly focused on methods’ performance assessment in terms of estimating concentrations. Szpiro et al^3^ found in a simulation study that improving the predictions in spatial LUR models did not always improve the health effect estimate as this was dependent on the Berkson-type of error and its differential impact on the exposure and health association as a component of the complex combination between classical and Berkson type-error in exposure assessment. Lee et al^21^ in a subsequent simulation study found that the validity and reliability of the health effect estimate can be greatly affected by the sampling of the monitor locations used to inform spatial LUR models, while Wang et al^22^ reported that decreases in forced vital capacity in relation to air pollution exposure were larger for LUR models with larger predictive ability in terms of holdout validation and cross-holdout validation. A recent review^2^ indicated that application of measurement error correction methods mainly in cohort designs, that applied a variety of exposure methods including spatial and spatiotemporal LUR and kriging methods, resulted in increases in effect estimates and their standard errors, which is in accordance with our simulation findings for long-term effects on background PM.

We recognize that the great majority of air pollution epidemiological studies follow either a time-series approach to investigate effects following short-term exposure or a cohort design for long-term exposure. As previous reports on measurement error investigated its effect under these designs, we aimed to expand the literature under a mixed modeling approach. In addition, the main objective of the STEAM project was the development of several exposure models for London and the optimal choice based on the best performance in terms of the effect estimation as assessed by simulations under this a-priori defined modeling approach. Hence we consider among the strengths of our study the comparison of several exposure assessment models in both the short- and long-term associations. Further the set up of the simulated surface incorporated both spatial and temporal complex covariances and correlations in contrast with most previous reports that focus on either aspect.^3,23^ We produced the validation data sets for our simulation on LUR and the machine learning algorithms using a 10% cross-validation to avoid including monitors incorporated in the methods in the setting of our simulation, although retrospectively that may be an overcorrection. Also our study was based in London where the number of fixed site monitors is large compared with other urban centers. The classification of our validation data and corresponding simulations by site type guards against driving the simulated “true” exposure at the centroid of the LSOA by this characteristic and further helps to identify if there is a weakness in terms of the ability of the model to predict for the one or the other of the site types.

Limitations include the lack of confounders in our epidemiological model and the uncertainty in mean bias over the simulations, which seems to be larger in our results compared with the gaseous analysis^1^. More importantly, the amount of measurement error in each exposure method may differ in other locations; hence our results are not directly transferable to other settings. We expect differential measurement error due to varying covariates informing the methods by location, although Vlaanderen et al^24^ suggest that the impact is modest in LUR providing the models perform well.

## Conclusions

Our simulations investigating the impact of the measurement error for PM_2.5_ and PM_10_ from various exposure assessment models on the health effect estimates support that the underestimation was larger when assessing long-term exposures. There were instances of nontrivial bias away from the null especially when roadside/kerbside monitoring sites were considered. Averaging of different exposure predictions performed best in almost all cases indicating that the integration of models to maximize performance is advisable.

## Conflicts of interest statement

The authors declare that they have no conflicts of interest with regard to the content of this report.

## Acknowledgments

We are grateful to the UK Met Office for provision of meteorological data, accessed through the Centre for Environmental Data Analysis (CEDA). We are also grateful to the UK Government and Local Authorities providing air pollution measurements used in this study, managed by King’s College London and Ricardo Energy and Environment.

## Supplementary Material


