# Supplementary material for: The impact of measurement error in modeled ambient particles exposures on health effect estimates in multilevel analysis: A simulation study
Source: Environ Epidemiol. 2020 May 27;4(3):e094. doi: 10.1097/EE9.0000000000000094 (PMC7319186; doi:10.1097/EE9.0000000000000094)
Supplement: Supplementary file 1 [file ee9-4-e094-s001.docx]

**eAppendix**

**The impact of measurement error in modelled ambient particles exposures on health effect estimates in multi-level analysis: a simulation study.**

Evangelia Samoli^1^, Barbara K. Butland^2^, Sophia Rodopoulou^1^, Richard W Atkinson^2^, Benjamin Barratt^3,4^, Sean D Beevers^3^, Andrew Beddows^3^, Konstantina Dimakopoulou^1^,Joel D Schwartz^5,6^, MahdiehDanesh Yazdi^5^, Klea Katsouyanni^1,4,7^

1. Department of Hygiene, Epidemiology and Medical Statistics, Medical School, National and Kapodistrian University of Athens, Athens, Greece.

2. Population Health Research Institute, St George’s, University of London, London, UK.

3. MRC Centre for Environment and Health, King’s College London, London, UK.

4. National Institute for Health Research Health Protection Research Unit (NIHR HPRU) in Health Impact of Environmental Hazards, King’s College London, London, UK.

5. Department of Environmental Health, Harvard School of Public Health, Boston, Massachusetts, USA.

6. Department of Epidemiology, Harvard School of Public Health, Boston, Massachusetts, USA.

7. School of Population Health and Environmental Sciences and MRC Centre for Environment and Health, King’s College London, London, UK.

**Table S1.**Baseline disease rates (C_3_) and concentration response function for short-term ($\beta_{1}$) and long-term $(\beta_{2})$ used in our simulations.

**Table S2.**Coverage probabilities and power for the simulations on PM_10_.

**Table S3.**Coverage probabilities and power for the simulations of the association between PM_2.5_ and all-cause mortality or cardiovascular admissions_._

[eAppendix references are listed at the end of this document]

**Table S1.**Baseline disease rates (C_3_) and concentration response function for short-term ($\beta_{1}$) and long-term $(\beta_{2})$ used in our simulations.

| Outcome | Baseline rate per LSOA per day  $exp(c_{3})$ | Pollutant | Concentration response function per 1 µg/m^3^ | |
| --- | --- | --- | --- | --- |
|  |  |  | Short-term exposure  $(\beta_{1})$ | Long-term exposure  $(\beta_{2})$ |
| All-cause Mortality | 0.0264§ | PM_10_ | 0.00032[1] | 0.00344[2] |
|  |  | PM_2.5_ | 0.00100[3] | 0.00686[4] |
| Cardiovascular hospital admissions | 0.0835¶ | PM_10_ | 0.00040[1] | 0.04055[5] |
|  |  | PM_2.5_ | 0.00091[6] | 0.00307[7] |

§Average death rate per LSOA per day in London in 2011 estimated using data from the Office for National Statistics.[8-9] ¶ Number of hospital admission per LSOA per day for the financial year 2011-2012 estimated using data from the Office for National Statistics,[8] and NHS Digital.[10]

**Table S2** Simulations’ results for the association between cardiovascular admissions and PM_10._

|  |  | Effect estimate for 10 μg/m^3^ increase in short-term exposure | | | | Effect estimate for 10 μg/m^3^ increase in long-term exposure | | | |
| --- | --- | --- | --- | --- | --- | --- | --- | --- | --- |
|  |  | $\hat{\beta_{1}}\times10$  $(se(\hat{\beta_{1}}) \times10)$ | Bias^a^  (%) | Coverage probability  (%) | Power  (%) | $\hat{\beta_{2}}\times10$  $(se(\hat{\beta_{2}}) \times10)$ | Bias^a^  (%) | Coverage Probability  (%) | Power  (%) |
| **Urban / Suburban** | Land Use Regression | 0.00466 (0.00233) | **16.6** | 95.1 | 51.5 | 0.04584 (0.08062) | -88.7 | 2.7 | 16.4 |
|  | Dispersion | 0.00388 (0.00144) | -2.7 | 95.3 | 77.7 | 0.19909 (0.09505) | -50.9 | 43.8 | 52.8 |
|  | Hybrid 1 | 0.00415 (0.00154) | **4.0** | 95 | 78.1 | 0.08338 (0.08245) | -79.4 | 5.9 | 23.7 |
|  | Hybrid 2 | 0.00428 (0.00159) | **7.3** | 95.1 | 76.4 | 0.20930 (0.09941) | -48.4 | 48.8 | 54.3 |
| **Roadside / Kerbside** | Land Use Regression | 0.00403 (0.00173) | **1.0** | 95.5 | 63.3 | 0.04116 (0.04997) | -89.8 | 0.0 | 15.5 |
|  | Dispersion | 0.00372 (0.00122) | -6.9 | 94.7 | 86.3 | 0.27562 (0.06056) | -32.0 | 43.3 | 98.4 |
|  | Hybrid 1 | 0.00370 (0.00124) | -7.3 | 94.3 | 84.7 | 0.10886 (0.05168) | -73.2 | 0.1 | 57.4 |
|  | Hybrid 2 | 0.00403 (0.00135) | **1.0** | 95.7 | 83.8 | 0.30194 (0.06531) | -25.5 | 62.2 | 98.9 |

The true effects considered were 0.0040 for short –term exposure and 0.4055 for long-term per 10 μg/m^3^ increase in PM_10_.

^a^Percent bias is highlighted in bold when positive (i.e. away from the null) rather than negative (i.e. towards the null).

**Table S3.**Simulations’ results for the association between cardiovascular admissions and PM_2.5._

|  | Model | Effect estimate for 10 μg/m^3^ increase in short-term exposure | | | | Effect estimate for 10 μg/m^3^ increase in long-term exposure | | | |
| --- | --- | --- | --- | --- | --- | --- | --- | --- | --- |
|  |  | $\hat{\beta_{1}}\times10$  $(se(\hat{\beta_{1}}) \times10)$ | Bias^a^  (%) | Coverage probability  (%) | Power  (%) | $\hat{\beta_{2}}\times10$  $(se(\hat{\beta_{2}}) \times10)$ | Bias^a^  (%) | Coverage Probability  (%) | Power  (%) |
| **Urban / Suburban** | Land Use Regression | 0.01054 (0.00371) | **16.3** | 92.4 | 81.0 | 0.00141 (0.12787) | -95.4 | 86.0 | 12.7 |
|  | Dispersion | 0.00722 (0.00206) | -20.3 | 84.9 | 93.7 | 0.00579 (0.13573) | -81.1 | 84.5 | 14.1 |
|  | Machine learning methods | 0.00815 (0.00234) | -10.0 | 93.5 | 93.8 | 0.00855 (0.16441) | -72.2 | 75.8 | 24.1 |
|  | Hybrid 1 | 0.00929 (0.00266) | **2.5** | 94.5 | 94.6 | 0.01411 (0.12849) | -54.1 | 86.4 | 13.5 |
|  | Hybrid 2 | 0.00900 (0.00258) | -0.7 | 95.1 | 94.1 | -0.00281 (0.15937) | -109.1 | 79.4 | 20.4 |
|  | Hybrid 3 | 0.00880 (0.00249) | -2.9 | 94.2 | 93.8 | 0.02418 (0.16757) | -21.3 | 84.3 | 16.8 |
| **Roadside Kerbside** | Land Use Regression | 0.00956 (0.00268) | **5.5** | 94.9 | 95.5 | 0.02895 (0.04308) | -5.8 | 46.6 | 54.6 |
|  | Dispersion | 0.00762 (0.00177) | -15.9 | 88.2 | 99.4 | 0.03886 (0.03989) | **26.5** | 57.5 | 46.5 |
|  | Machine learning methods | 0.00884 (0.00200) | -2.4 | 95.2 | 99.5 | 0.05307 (0.04415) | **72.7** | 43.0 | 61.8 |
|  | Hybrid 1 | 0.00876 (0.00203) | -3.3 | 94.8 | 99.4 | 0.03658 (0.04031) | **19.0** | 51.4 | 49.7 |
|  | Hybrid 2 | 0.00947 (0.00220) | **4.5** | 94.8 | 99.2 | 0.04896 (0.04084) | **59.3** | 50.7 | 53.1 |
|  | Hybrid 3 | 0.00944 (0.00212) | **4.2** | 95.3 | 99.7 | 0.03886 (0.04580) | **26.5** | 63.9 | 40.5 |

The true effects considered were 0.0091 for short –term exposure and 0.0307 for long-term per 10 μg/m^3^ increase in PM_2.5_.

^a^Percent bias is highlighted in bold when positive (i.e. away from the null) rather than negative (i.e. towards the null).

**References**

[1] Katsouyanni K, Samet JM. Air pollution and Health: A European and North AmericanApproach (APHENA). Health Effect Institute Report, Number 142, October 2009, p28 and 43, Tables 11 and 32. [Lag 1, partial autocorrelation function, natural spline model]*.*

[2] Hoek G, Krishnan RM, Beelen R, Peters A, Ostro B, Brunekreef B, Kaufman JD. Long-term air pollution exposure and cardio- respiratory mortality: a review. Environ Health 2013;12(1):43.

[3] Anderson HR, Atkinson RW, Bremner SA, Carrington J, Peacock J. Quantitative systematic review of short term associations between ambient air pollution (particulate matter, ozone, nitrogen dioxide, sulphur dioxide and carbon monoxide), and mortality and morbidity. Report to Department of Health revised following first review, June 2007, p69, Table 4.2a. URL: <https://www.gov.uk/government/publications/quantitative-systematic-review-of-short-term-associations-between-ambient-air-pollution-particulate-matter-ozone-nitrogen-dioxide-sulphur-dioxide-and-carbon-monoxide-and-mortality-and-morbidity>

[4] Faustini A, Rapp R, Forastiere F. Nitrogen dioxide and mortality: review and meta-analysis of long-term studies. EurRespir J 2014;44(3):744-53.

[5] Katsoulis M, Dimakopoulou K, Pedeli X, Trichopoulos, Gysparis A, Trichopoulou A, Katsouyanni K. Long-term exposure to traffic-related air pollution and cardiovascular health in a Greek cohort study. Sci Total Environ 2014;490;934-940. (Table 2)

[6] WHO. Health risk of air pollution in Europe - HRAPIE project. Recommendations for concentration–response functions for cost–benefit analysis of particulate matter, ozone and nitrogen dioxide. WHO Regional Office for Europe Report, 2013, page 6.

[7] Kloog I, Coull BA, Zanobetti A, Koutrakis P, Schwartz JD. Acute and chronic effects of particles on hospital admissions in New-England. PLoS One 2012;7(4):e34664. (Table 3)

[8] Office for National Statistics. 2011 Census: Usual residents by resident type, and population density, number of households with at least one usual resident and average household size, Output Areas (OAs) in London.https://www.ons.gov.uk /peoplepopulationandcommunity/ populationandmigration/ populationestimates/datasets/ 2011censuspopulationandhouseholdestimatesforwardsandoutputareasinenglandandwales. Accessed 22 Aug 2017. The data are © Crown Copyright 2012, licensed under the Open Government Licence v3.0.<http://www.nationalarchives.gov.uk/doc/open-government-licence/version/3/>.

[9]Office for National Statistics‚ National Records of Scotland‚ Northern Ireland Statistics and Research Agency. Mortality Statistics: Deaths registered by area of usual residence, 2011 registrations. [https://www.ons.gov.uk/peoplepopulationandcommunity/ birthsdeathsandmarriages/deaths/datasets/deathsregisteredbyareaofusualresidenceenglandandwales](https://www.ons.gov.uk/peoplepopulationandcommunity/%20birthsdeathsandmarriages/deaths/datasets/deathsregisteredbyareaofusualresidenceenglandandwales). Accessed 21 Aug 2017. The data are © Crown Copyright 2013, licenced under the Open Government Licence (OGL) v3.0. <http://www.nationalarchives.gov.uk/doc/open-government-licence/version/3/>.

[10] NHS Digital. Hospital Episode Statistics Admitted Patient Care - England, 2011-12: Provider-level analysis. Table A: “Headline figures for England, SHA and individual provider (2011-2012)” and Table E: “Finished admission episodes by primary diagnosis chapter for England, SHA and individual provider”. <https://digital.nhs.uk/data-and-information/publications/statistical/hospital-admitted-patient-care-activity/hospital-episode-statistics-admitted-patient-care-england-2011-12>. Source: Hospital Episode Statistics, HES. The Health and Social Care Information Centre. Information from NHS Digital, licenced under the current version of the Open Government Licence. Accessed 28^th^ August 2018.
